# Low pH, High Stakes: A Narrative Review Exploring the Acid-Sensing GPR65 Pathway as a Novel Approach in Renal Cell Carcinoma

**DOI:** 10.3390/cancers17233883

**Published:** 2025-12-04

**Authors:** Michael Grant, Barbara Cipriani, Alastair Corbin, David Miller, Alan Naylor, Stuart Hughes, Tom McCarthy, Sumeet Ambarkhane, Danish Memon, Michael Millward, Sumanta Pal, Ignacio Melero

**Affiliations:** 1 St Bartholomew’s Hospital, London EC1A 7BE, UK; 2 Weatherden Limited, London WC1V 6DF, UK; 3 Pathios Therapeutics Limited, Oxford OX2 6HJ, UK; 4 Molecule to Medicine, Oxford OX1 4PS, UK; 5 School of Medicine, University of Western Australia and Linear Clinical Research, Perth, WA 6009, Australia; 6 City of Hope Comprehensive Cancer Center, City of Hope, Duarte, CA 91010, USA; 7 Department of Immunology and Immunotherapy, Clinica Universidad de Navarra, 31008 Pamplona, Spain

**Keywords:** renal cell carcinoma, tumour microenvironment, tumour acidosis, immune checkpoint inhibitors, GPR65, carbonic anhydrase IX, immunotherapy, treatment resistance

## Abstract

Renal cell carcinoma (RCC) is a biologically heterogeneous malignancy accounting for 3% of adult cancers globally. Despite advances in immune checkpoint inhibitors (ICIs) and vascular endothelial growth factor (VEGF)-targeted therapies, durable disease control remains elusive for many patients. Increasing evidence implicates the acidic tumour microenvironment (TME) as a critical mediator of RCC progression, immune evasion, and therapeutic resistance. Solid tumours, including RCC, exhibit reversed pH gradients, characterised by acidic extracellular (pH 6.2–6.9) and alkaline intracellular conditions. This dysregulation arises from enhanced glycolysis, hypoxia-driven lactate accumulation, and the overexpression of pH-regulating enzymes such as carbonic anhydrase (CA9). Acidic TMEs impair cytotoxic T-cell and NK-cell activity, promote tumour-associated macrophage (TAM) polarisation towards an immunosuppressive phenotype, and upregulate alternative immune checkpoints. These mechanisms collectively undermine ICI efficacy and contribute to primary and secondary treatment resistance. Proton-sensing G-protein-coupled receptors (GPCRs), notably GPR65, have emerged as pivotal mediators linking extracellular acidosis to immune dysfunction. Preclinical studies demonstrate that GPR65 antagonists restore anti-tumour immune activity by reversing acidosis-driven immunosuppression and enhancing antigen processing. In RCC models, selective GPR65 inhibitors have shown the ability to reduce immunosuppressive cytokine IL-10 production, induce immunoproteasome activation, and synergise with anti-PD-1 therapy. The first-in-class GPR65 inhibitor, PTT-4256, is now under evaluation in the Phase I/II RAISIC-1 trial (NCT06634849) in solid tumours, including RCC. Targeting acid-sensing pathways represents a novel and promising therapeutic strategy in RCC, aiming to remodel the TME and overcome ICI resistance. Integrating GPR65 inhibition with existing immunotherapies may define the next era of RCC management, warranting continued translational and clinical investigation.

## Simple Summary

Renal cell carcinoma is a type of kidney cancer that remains difficult to treat, even with modern immunotherapy and targeted drugs. One important reason for treatment failure is that these tumours create an unusually acidic environ-ment around themselves. This acidic setting weakens the body’s immune cells, helps cancer cells avoid detection, and makes therapies less effective. Recent research has identified a sensor on immune cells, called GPR65, that becomes activated in acidic conditions and contributes to this immune suppression. Blocking GPR65 has been shown in early laboratory studies to restore the ability of immune cells to recognise and attack cancer. New medicines that target this pathway are now being tested in clinical trials. This research aims to understand how targeting acidity-sensing mech-anisms could improve responses to immunotherapy and lead to more effective treatments for people with kidney cancer.

## 1. Introduction

### 1.1. Epidemiology of Renal Cell Carcinoma

There are over 400,000 new cases of renal cell carcinoma (RCC) diagnosed per year globally—accounting for 3% of all adult malignancies [1,2]. Despite advances in treatment and diagnosis in recent years, RCC is responsible for 175,000 deaths annually, with the number of diagnoses set to increase in coming years [3]. RCC rates have almost doubled since the 1990s, making it the 12th most common tumour type globally [2,3,4]. These increases are driven by the growing prevalence of risk factors more commonly seen in developed countries (e.g., smoking, obesity, hypertension, and chronic kidney disease). However, similar trends are emerging in developing countries as a result of globalisation that will exacerbate the global rise in RCC incidence [2,5]. Over 50% of RCCs are now detected incidentally in patients without specific symptomology, some of whom skew younger than the traditional median age of diagnosis for RCC [3,5,6]. Less-advanced disease may present with microscopic haematuria, non-specific abdominal pain, and/or unexplained constitutional symptoms. Rarely, paraneoplastic syndromes (e.g., hypertension, hypercalcemia, and polycythaemia) can also precede RCC diagnosis [7]. Over 95% of RCCs are considered sporadic in aetiology, with the remainder mainly resulting from rare hereditary conditions—such as Von Hippel–Lindau (VHL), Birt–Hogg–Dubé, and tuberous sclerosis syndromes [8,9]. The introduction of immunotherapy—particularly immunotherapy and anti-angiogenesis-targeted combinations—to the standard of care for RCC has improved outcomes, yet the five-year survival for metastatic disease remains poor [10]. Moreover, there is an observable disparity in the mortality-to-incidence ratio of RCC in less developed settings—further highlighting the need for more effective and equitable treatment options for patients [2].

### 1.2. Renal Cell Carcinoma Subtypes

RCC encompasses the vast majority of cancers arising in the kidney (>90%) and, specifically, describes tumours with epithelial histology arising from the renal cortex, particularly the parenchymal epithelial cells within the proximal tubule [11]. Rarer, distinct forms of kidney cancer include transitional cell carcinoma of the renal pelvis—which behaves like urothelial cancer—and renal sarcoma [12,13]. Clear-cell (ccRCC) subtypes combined with the two most common ‘non-clear-cell’ subtypes—papillary (pRCC) and chromophobe (chRCC)—account for virtually all RCC diagnoses. These all arise from the renal tubular epithelium but exhibit distinct histologic, molecular, and clinical features that can influence prognosis and therapeutic strategies [11,12,14]. In 2022, the WHO classification of genitourinary tumours was updated and placed an increased focus on molecular profiling when defining subtypes of RCC [15]. While this retained these three main subtypes, several other subtypes (e.g., oncocytoma, collection duct, and medullary) are described. However, these constitute only a fraction of the cases [12,16].

ccRCC is the most common subtype of RCC, being the cause of eight out of every ten cases. It is characterised histologically by malignant epithelial cells with clear cytoplasm due to glycogen and lipid accumulation and a rich vascular network [17]. Molecularly, ccRCC is strongly linked to loss-of-function mutations in the VHL gene and frequent chromosome 3p loss; PBRM1, BAP1, and SETD2 mutations are also implicated and influence prognosis. Clinically, ccRCC is more aggressive than other subtypes, demonstrating higher metastatic potential at diagnosis and greater resistance to chemotherapy and radiotherapy [12,17]. Prognosis is closely tied to stage at diagnosis, with localised tumours having a favourable outlook while patients with metastatic ccRCC at detection having a 5-year survival rate as low as 10% [12,18].

Of the ‘non-clear-cell’ variants, pRCC accounts for 15% of all RCC cases and was previously described as including two distinct forms: type 1, often MET-driven and indolent, and type 2, associated with FH and CDKN2A mutations and a more aggressive course. However, following the 2022 WHO updates, it is simply referred to as papillary but with a greater focus on mutational analysis guiding treatment [15]. Targeted therapies—particularly MET inhibitors such as cabozantinib—have shown effect in selected patients [19]. chRCC is relatively rare (approximately 5% of RCC cases) and typically follows a more indolent clinical course with lower metastatic potential—although tumours with sarcomatoid features may behave more aggressively. In contrast to ccRCC and pRCC, it arises from collecting duct intercalated cells and demonstrates a characteristic histology (pale, eosinophilic polygonal cells with perinuclear halos that mimic the typical appearance of plant cells) and widespread chromosomal losses [20].

### 1.3. Treatment Options for Renal Cell Carcinoma

Localised RCC, regardless of subtype, that has not spread beyond the kidney is treated by surgical resection—either partial or radical nephrectomy, depending on the tumour size and location—with curative intent [21]. Non-surgical techniques, such as radiofrequency ablation, cryoablation, and stereotactic radio-ablation, may be considered for the treatment of small (<3 cm) or bilateral renal lesions, as well as for patients who are too frail for surgery [22]. The frequency of surveillance imaging following surgery is based on prognostic risk scores (e.g., Lebovich), with features like size, histological grade, and necrosis generally conferring a higher chance of disease recurrence [23]. There is currently a paucity of evidence for neoadjuvant therapy in RCC; however, there is growing support for the use of immune checkpoint inhibitors (ICIs) in the adjuvant setting. The KEYNOTE-564 study randomised post-surgical patients with intermediate-to-high-risk disease to either adjuvant pembrolizumab or placebo. This showed a significant improvement in overall survival and has resulted in the approval of pembrolizumab in this setting for patients with a high risk of recurrence [16].

The treatment landscape for non-operable and metastatic RCC has undergone several significant transformations over the past four decades [16,24]. While there are some nuances—such as the use of MET and mTOR inhibitors in pRCC and chRCC, respectively—all RCC subtypes are typically treated with agents first approved in ccRCC [18,19,25]. Cytokine therapy with interferon-α (IFN-α) and interleukin-2 (IL-2) dominated treatment from the late 1980s until 2007, when the introduction of VEGF tyrosine kinase inhibitors (TKIs) revolutionised RCC treatment. Sunitinib became the first-line standard of care after demonstrating superiority against cytokine therapy, with several more VEGF TKIs being authorised since [26]. The current era began with immune checkpoint inhibitors, initially with nivolumab monotherapy approved in 2015 as a second-line therapy after VEGF-TKI failure [27]. The landmark CheckMate 214 trial established nivolumab plus ipilimumab as superior to sunitinib in intermediate and poor-risk patients, which was followed by the KEYNOTE-426 study heralding first-line ICI-TKI combination therapy [28,29]. Additionally, during this shift in the standard of care, the evidence turns against the inclusion of cytoreductive nephrectomy in metastatic disease, with no benefit being seen for patients receiving TKI therapy [30]. Current first-line recommendations now centre on ICI-based combinations, with pembrolizumab plus axitinib, avelumab plus axitinib, pembrolizumab plus lenvatinib, and cabozantinib plus nivolumab all being approved combinations [16,29].

Outcomes for patients with advanced RCC have markedly improved with contemporary treatments, yet a subset of patients do not derive benefit at all. Moreover, many of those that initially achieve complete or partial remission ultimately experience disease progression due to acquired resistance [31]. Possible contributors to primary and secondary refractory RCC include hypoxia-driven angiogenesis, intra- and intertumoral genetic heterogeneity, immunosuppressive cellular infiltrates (such as regulatory T-cells [T Regs], tumour-associated macrophages [TAMs], and myeloid-derived suppressor cells [MDSCs]), alterations in tumour cell metabolic pathways, expression of alternative immune checkpoints (e.g., LAG-3, VISTA, TIGIT, and TIM-3), and host factors such as the microbiome [32,33]. Among the factors that may explain the limitations in treatment efficacy seen in RCC—and other solid tumours—is the acidic tumour microenvironment (TME).

### 1.4. The Role of the Acidic Tumour Microenviroment in Renal Cell Carcinoma

The accumulation of acidic compounds within the TME represents a fundamental characteristic of solid tumours, arising from enhanced glycolysis, hypoxia-driven lactate accumulation, and the overexpression of pH-regulating enzymes such as carbonic anhydrase IX (CA9) [34,35]. By amplifying processes such as epithelial–mesenchymal transition (EMT), immune suppression, altered drug uptake, and angiogenesis, low extracellular pH in the TME plays a role in multiple hallmarks of cancer progression and resistance in RCC [36]. Extracellular acidosis promotes sustained proliferative signalling by enabling cell cycle progression, while simultaneously conferring resistance to apoptosis through the inhibition of caspase activation [37,38,39]. The acidic TME drives invasion and metastasis via multiple mechanisms, including the activation of pH-dependent proteases—such as matrix metalloproteinases (MMPs) and cathepsins—that degrade the extracellular matrix, the induction of EMT, and the promotion of a stem-like phenotype in cancer cells [40,41,42]. Furthermore, tumour acidity facilitates immune evasion by impairing cytotoxic T-cells and natural killer (NK) cell function while promoting immunosuppressive cell populations—including TAMs and T Regs—through lactate-mediated metabolic reprogramming. For example, acidic extracellular conditions suppress the IL-2-mTORC1-c-Myc axis in CD8+ T-cells, reducing proliferation, cytotoxicity, and metabolic fitness [43,44]. Moreover, it has been demonstrated that acidosis in the TME—alongside the accumulation of lactate—upregulate PD-L1 on cancer cells via the activation of STAT1 (IFN-γ/eIF4F pathway) and STAT3, facilitating immune evasion [45,46,47]. Hypoxia-induced angiogenesis is further augmented by acidosis through the stabilisation of the hypoxia-inducible factor (HIF) and upregulation of the vascular endothelial growth factor (VEGF), creating a dysfunctional vasculature that perpetuates the hostile TME and that also impacts the penetration of immune cells and therapeutics into tumours [37,48,49,50]. Understanding these pH-driven mechanisms offers new opportunities to overcome established barriers to effective cancer control [51].

In RCC, the acidic TME is particularly relevant due to the VHL gene inactivation observed in the majority of cancers that arise from the kidney. A loss of VHL function results in the constitutive stabilisation of HIFα subunits—independent of the presence of hypoxia—leading to the transcriptional upregulation of CA9 and other pH-regulatory machinery that maintain the reversed pH gradient [35,52,53]. This state undermines therapeutic efficacy through multiple mechanisms: impaired drug uptake of weakly basic chemotherapeutic agents, radiotherapy resistance via the inhibition of DNA damage checkpoints, and the promotion of lysosomal sequestration of TKIs (reducing their bioavailability at target sites and, thus, treatment efficacy) [33,54,55]. Low pH also facilitates resistance to ICIs by enhancing PD-L1 expression on cancer cells through acidosis-mediated STAT1 activation, suppressing effector T-cell function, and fostering an immunosuppressive TME dominated by exhausted T-cells and pro-tumourigenic macrophages [33,56,57]. The clinical relevance of this is supported by real-world evidence that has shown an association between the co-infiltration of M2 TAMs and T Regs with poor outcomes in RCC patients [58]. Collectively, the acidic TME in RCC represents a multifaceted driver of oncogenesis, disease progression, and therapeutic resistance—highlighting the potential therapeutic benefits of targeting components of acid regulation in RCC.

### 1.5. Proton-Sensing G-Protein-Coupled Receptors as a Novel Therapeutic Target in Renal Cell Carcinoma

Proton-sensing G-protein-coupled receptors (GPCRs) play crucial roles in both innate and adaptive immune cell function within acidic TMEs. In particular, GPR65 emerges as an important proton-sensing receptor in cancer biology, exhibiting both tumour-promoting and tumour-suppressive functions depending on cellular context and cancer type [59,60,61,62,63]. Pan-cancer analysis reveals variable GPR65 expression patterns across different tumour types, with significant correlations between expression levels and patient prognosis [64]. Analyses of TME RNAseq datasets have also established that monocytes, macrophages, CD8+ T-cells, and NK cells are the most frequent GPR65-expressing tumour-infiltrating cells—all known to play critical roles in cancer immunity [65]. Macrophage polarisation has been shown to be significantly influenced by proton-sensing GPR65 activation, with activation fostering immunosuppressive phenotypes [66,67]. T-cell function is also impaired via GPR65 signalling, with experimental data demonstrating that GPR65 activity significantly inhibited antigen-specific, CD8+ T-cell cytotoxic activity against target cancer cells [65]. Both of these examples are particularly relevant to RCC, in which immunosuppressive TAMs and impaired CD8+ function are associated with disease progression and treatment resistance [68,69]. Location-biased activation of GPR65 allows for compartment-specific signalling, with different subcellular localisations producing distinct functional outcomes. GPR65 coupling to Gαs-cyclic AMP (cAMP) pathways mediates many of its anti-inflammatory and immunoregulatory effects, while coupling to other G-protein subtypes may produce pro-neoplastic effects [62,63,70,71].

### 1.6. Review Methods

This is a narrative review that synthesises published evidence on tumour acidosis and GPR65 in RCC; it is not a systematic review or meta-analysis. The authors searched PubMed/MEDLINE for English-language articles from 2005 to 2025 using combinations of the following terms: renal cell carcinoma, acidic TME, tumour acidosis, carbonic anhydrase IX/CA9, proton-sensing GPCRs, GPR65, GPR68, GPR4, immune evasion, immunotherapy resistance, and checkpoint inhibitors. We also screened ClinicalTrials.gov and relevant major-meeting abstracts (e.g., ASCO, ESMO, SITC, and AACR). We included primary research, high-quality reviews, and consensus/position papers relevant to RCC or tumour acidosis biology. We prioritised peer-reviewed sources and identified whether data were independent third-party or sponsor-generated. As this is a narrative synthesis, the authors did not perform a formal, quantitative risk-of-bias assessment; instead, we weighted evidence by study design, replication, and disease relevance (RCC-specific where available). Any proprietary or unpublished findings (e.g., Pathios’ Supplemental data and models) are clearly labelled and used to illustrate mechanistic plausibility rather than to draw definitive conclusions.

## 2. Acidic Tumour Microenvironments in Solid Tumours

The acidic TME represents one of the most distinctive and clinically relevant hallmarks of solid tumours, fundamentally distinguishing malignant tissues from their normal counterparts [51]. Normal tissues maintain extracellular pH values between 7.2 and 7.4, tightly regulated by the excretion of volatile and non-volatile acids produced in the body. However, solid tumours exhibit markedly acidic extracellular environments (with pH values ranging from 6.2 to 6.9) arising from a combination of metabolic rewiring as well as a hypoxic microenvironment that favours the accumulation of glycolytic and non-volatile acids [72]. This creates a reversed pH gradient, wherein intracellular pH is comparatively alkaline to the extracellular space. This dysregulated acid–base homeostasis serves as a byproduct of aberrant tumour metabolism, but also as a driver of cancer progression, treatment resistance, and immune evasion [34,35]. RCC—particularly ccRCC—exemplifies these extreme metabolic and molecular adaptations that underpin extracellular acidification in the TMEs of solid tumours. RCC arises in a unique context of renal tubular cell biology where the constitutive loss of VHL function drives HIF activation, which in turn upregulates key effectors such as CA9, monocarboxylate transporters (MCTs), and lipid metabolic enzymes.

### 2.1. pH Detection and Normal Physiology of Acid–Base Balance

In healthy tissues, acid–base homeostasis is meticulously maintained through the coordinated function of multiple physiological systems. Systemic regulation involves the bicarbonate buffer system (HCO_3_^−^/CO_2_), renal acid excretion, and pulmonary CO_2_ elimination, maintaining blood pH within the narrow range of 7.35–7.45. The kidney serves as the primary organ for long-term acid–base regulation, with proximal tubules reabsorbing approximately 80% of filtered bicarbonate and distal nephron segments fine-tuning acid excretion. Cellular buffering systems include phosphate buffers, protein histidine residues, and haemoglobin, which provide immediate pH stabilisation during acid–base perturbations [73].

At the cellular level, the key processes involved in sustaining pH homeostasis include the active transport of acid–base equivalents (such as H^+^ and HCO_3_^−^) via membrane-bound transporters: sodium–hydrogen exchangers, sodium–bicarbonate cotransporters, chloride–bicarbonate exchangers, and vacuolar ATPases [74,75]. These transporters regulate intracellular pH by mediating the extrusion, reabsorption, or exchange of protons and bicarbonate ions in response to metabolic activity and extracellular cues, collectively maintaining an intracellular pH of around 7.2 [73,76]. Cells sense and respond to changes in extracellular pH through a sophisticated network of molecular sensors and signalling pathways—these include proton-sensing GPCRs, acid-sensing ion channels (ASICs), and soluble adenylate cyclase (sAC).

Proton-sensing GPCRs—notably GPR4, GPR65 (also known as T-cell death-associated gene 8, TDAG8), and GPR68 (also known as ovarian cancer G-protein-coupled receptor 1, OGR1)—are transmembrane receptors that detect extracellular acidification [77,78]. These receptors possess critical extracellular histidine residues that change their protonation state as pH drops, leading to conformational changes and the activation of intracellular signalling pathways [79]. The different GPCR subtypes have varied roles: GPR4 and GPR68 tend to be pro-inflammatory, while GPR65 shows mainly anti-inflammatory activities. Upon activation by extracellular acidosis, these receptors regulate diverse functions such as angiogenesis, immune cell modulation, and cell proliferation, as well as responses to inflammation and pain [78,80]. The proton-sensing GPCRs exhibit heterogeneity in G-protein coupling and secondary messenger production, distinguishing them from many conventional GPCRs through their capacity for promiscuous G-protein interactions [80]. GPR4 couples primarily to Gαs to stimulate adenylyl cyclase and cAMP production, though it can also activate Gαq/11, Gα12/13, and Gαi pathways depending on context. Similarly, GPR65 predominantly signals through Gαs and Gα13, activating the cAMP-protein kinase A (PKA) cascade that drives its immunosuppressive effects, with emerging evidence suggesting additional signalling complexity via sAC and endosomal compartments [65,81,82]. In contrast, GPR68 couples most commonly to Gαq/11 to activate phospholipase C, generating IP3, diacylglycerol, and intracellular calcium mobilisation; though it retains the ability to activate Gαs-dependent cAMP production under certain conditions. Their role is evident in many physiological and pathological processes, including cancer, ischaemia, and chronic inflammation [77,80].

ASICs constitute a distinct family of ligand-gated ion channels widely expressed in neuronal and non-neuronal tissues. Upon extracellular acidification, ASICs open to allow the influx of sodium (and occasionally calcium) ions, resulting in rapid changes in membrane potential and excitability [74]. These channels are primary mediators of acid-induced pain and play key roles in nociception, synaptic transmission, and neurodegeneration [83]. ASICs are increasingly found ectopically expressed in various carcinomas and some brain tumours, but the mechanistic understanding of ASIC function in cancer remains unknown [49].

sAC operates inside the cell as a sensor of bicarbonate and thus, indirectly, of intracellular pH. Unlike classical membrane-bound adenylate cyclases, sAC is activated by bicarbonate ions and can respond rapidly to cellular acid–base changes [84,85]. When activated, sAC raises intracellular cAMP levels, which affects numerous downstream targets, including the regulation of proton secretion and energy metabolism. Cells lacking sAC exhibit higher MAP kinase (MAPK) pathway activity. This may be complemented by oncogenes (such as SV40 large T antigen or human papillomavirus E6), supporting neoplastic transformation and tumour growth [84].

### 2.2. Pathophysiology of Tumour Acidification

The development of acidic conditions in solid tumours results from the convergence of metabolic reprogramming, vascular abnormalities, and cellular adaptation mechanisms [35,51]. The Warburg effect describes the preferential metabolism of glucose by glycolysis, even under aerobic conditions. In these circumstances, lactate dehydrogenase A (LDHA) preferentially converts pyruvate to lactate to rapidly produce ATP; this contrasts with Lactate dehydrogenase B (LDHB), which has a higher affinity for lactate and prefers converting it back to pyruvate when oxygen is present, supporting aerobic metabolism [86]. This effect generates substantial quantities of lactate and protons, leading to a metabolic shift as acid loads exceed normal cellular buffering capacity—inducing acid extrusion mechanisms [87,88,89]. Hypoxic conditions prevalent in poorly vascularised tumour regions further exacerbate lactate production while concurrently impairing perfusion-dependent acid clearance, demonstrating the synergistic relationship between hypoxia and acidosis in the TME [88,90,91].

A number of cellular apparatuses are coopted in cancer to nurture an acidic TME. CA9 is a transmembrane zinc metalloenzyme that plays a central role in tumour acidification by catalysing the reversible hydration of CO_2_ to bicarbonate and protons at the cell surface [53]. Its expression is tightly regulated in normal tissues, but it is known to be overexpressed in solid tumours, including RCC [50]. CA9 functions as an extracellular pH thermostat—sensing and responding to pH changes—actively maintaining acidic extracellular conditions by re-importing bicarbonate whilst leaving resultant protons outside the membrane [92]. The sodium–hydrogen exchanger isoform 1 contributes significantly to the reversed pH gradient by actively extruding intracellular protons in exchange for sodium ions, maintaining relatively alkaline intracellular conditions while acidifying the extracellular space [89]. Vacuolar ATPases and MCTs further facilitate acid extrusion, with MCTs specifically transporting lactate and associated protons across the cell membrane [93]. These work in concert to cultivate an acidic TME that supports cancer cell survival while creating hostile environments for normal cells and immune effectors [35,51].

Acidification patterns differ between primary tumours and metastatic lesions, reflecting the evolutionary pressures and microenvironmental constraints of different anatomical sites. Primary tumours develop acidosis gradually as they outgrow their vascular supply, with central necrotic regions displaying the most severe acidification while peripheral regions maintain moderate acidity [94]. Metastatic lesions often establish acidic microenvironments more rapidly, utilising the acid-modulating machinery that has already been upregulated during the earlier neoplastic processes of the primary tumour [40,51,88]. The “seed and soil” hypothesis suggests that acidic conditions in potential metastatic sites may favour circulating tumour cell implantation and growth, creating a positive selection pressure where acidosis both promotes metastasis and supports metastatic growth [91].

In RCC, acidic microenvironments develop through pathways that reflect both the unique biology of renal epithelial cells and the characteristic genetic alterations of this malignancy [36]. CA9 is constitutively overexpressed in ccRCC due to VHL gene loss, with 88.2% of tumours showing CA9 upregulation independent of VHL mutation status. This overexpression occurs in over 90% of ccRCC cases but is rarely found in normal renal tissues—making CA9 both a diagnostic marker and a therapeutic target [41,95]. The metabolic reprogramming in RCC involves enhanced glycolysis and altered fatty acid metabolism, contributing to acid production and creating conditions that support immune evasion [36,96]. Recent multi-omics analyses reveal that CA9 overexpression exerts effects beyond the canonical, pH-regulatory functions of this enzyme. Spatiotemporal mapping of the RCC TME demonstrates that CA9 activity is most pronounced at the hypoxic, nutrient-deprived core, where it exploits the synergistic presence of low oxygen and high acidity to promote invasion and immune escape [53,92,97]. In both primary and metastatic RCC, the interplay of hypoxia and acidity creates distinct regions wherein a dynamic regulation of CA9, MCT 1 and 4, and other transporters are seen—delineating areas of high glycolytic output and lactate export from those where fatty acid oxidation and lipid droplet accumulation predominate [97,98]. Lipid metabolism has emerged as a crucial contributor to RCC acidification. ccRCC tumours are uniquely characterised by cytoplasmic lipid droplet accumulation driven by the HIF-mediated upregulation of fatty acid synthesis enzymes, increased lipid uptake, and mitochondrial dysfunction reducing beta-oxidation efficiency. This rewired lipid metabolism increases proton production and can support energy needs via non-canonical metabolic pathways, while also impacting redox balance and the generation of immunosuppressive lipid mediators [57,98,99,100]. A downregulated urea cycle and glutamine metabolism further exacerbate intracellular acidosis, promoting survival under metabolic stress. MCT1 and MCT4, upregulated in RCC, efficiently shuttle lactate and protons into the TME, establishing a positive feedback loop with CA9 that reinforces extracellular acidosis and contributes to multidrug resistance [49,53,92]. Together, these adaptations create heterogenous, metabolically distinct geographical regions within RCC lesions marked by variable acidity and hypoxia—with high CA9 expression often coinciding with immunosuppressive areas lacking effector immune cells [57,96,97,101].

### 2.3. Low pH in Cancer Progression and Treatment Resistance

**Figure 1:**
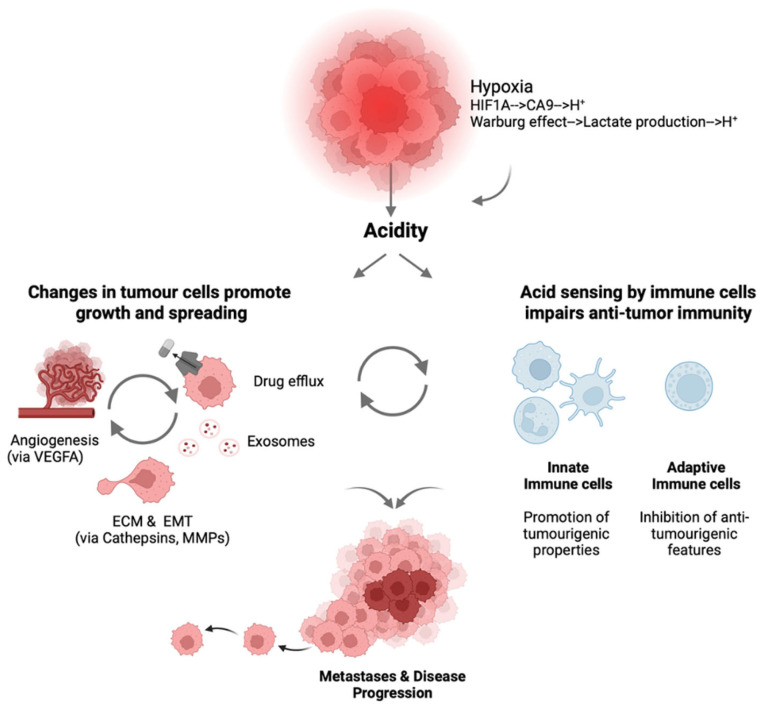
Schematic summarising the effects of acidity in the tumour microenvironment. HIF1A, hypoxia-inducible factor 1 subunit alpha; CA9, carbonic anhydrase IX; H^+^, hydrogen ion; VEGFA, vascular endothelial growth factor alpha; ECM, extracellular matrix; EMT, epithelial–mesenchymal transition; and MMP, matrix metalloproteinase. Created in https://Biorender.com.

The acidic TME actively promotes multiple hallmarks of cancer progression through direct and indirect mechanisms. There is strong evidence across tumour types—particularly RCC, head and neck, breast, pancreatic, and prostate—associating the pathophysiology induced by the low pH in the TME with a poor prognosis [49,51,102,103]. Invasion and metastasis are enhanced by the acid-induced activation of proteolytic enzymes, including cathepsins and matrix metalloproteinases that degrade extracellular matrix components [33,36,51]. EMT is promoted by chronic acidosis (e.g., through TGF-β2/miR-7 signalling pathways and the downregulation of miR-193b-3p), enabling cellular dedifferentiation. Extracellular vesicle release is significantly enhanced under acidic conditions, promoting intercellular communication, genetic instability, and resistance mechanisms (e.g., drug efflux pumps) [40,104,105]. Angiogenesis is stimulated by acidic conditions through the stabilisation of HIFs and upregulation of VEGF signalling pathways [51]. Cancer stem cell properties are enhanced in acidic microenvironments, with acidosis promoting stemness markers and increasing further tumorigenic potential [88]. Of all these hallmarks, immune evasion represents a critical consequence of tumour acidification with regard to both disease progression and treatment resistance [Figure 1] [32,33].

Low pH in the TME orchestrates a multifaceted immunosuppressive landscape, affecting both immune cell infiltration and function through several distinct mechanisms. Firstly, acidic conditions impair the activity and expression of adhesion molecules (e.g., integrins and selectins) on endothelial and immune cells, restricting travel through cancerous tissue and, thus, limiting effective immunosurveillance [49,106,107]. Secondly, extracellular acidosis induces structural changes in the extracellular matrix, alters tissue stiffness, and disrupts chemokine gradients; these changes block the chemotactic migration of cytotoxic lymphocytes and further hinder immune cell infiltration [52,94,106]. Thirdly, the low glucose and high lactate concentrations resulting from increasing acidity impede effector immune cell function and proliferation. Cytotoxic T-cells are particularly reliant on glucose metabolism while, in contrast, T Regs are bolstered by these conditions through the stabilisation of FoxP3—thus favouring T Reg survival and expansion over effector T-cells within the TME [40,108,109,110]. Finally, the increasing dominance of T Regs in the TME perpetuates local immunosuppression through negative feedback. T Reg-driven cytokine release and immune checkpoint activity then further suppress infiltrating effector cells—cementing an immunosuppressive niche for tumour cells to grow unencumbered [68,94,110].

Low pH contributes to treatment resistance through multiple interconnected pathways that affect both cytotoxic and targeted therapies. Drug efflux pumps (e.g., P-glycoprotein) are upregulated in response to acidic stress actively reducing intracellular drug concentrations, while ion trapping of weak base chemotherapeutic agents occurs in acidic extracellular environments, reducing drug uptake and efficacy [35,111]. Autophagy activation in normal cells under acidic stress provides a cytoprotective niche, helping cancer cells survive therapeutic interventions that use stimulated immune responses [36,72]. Immunotherapy resistance is particularly problematic in acidic tumours, where low pH impairs T-cell infiltration, reduces cytotoxic function, and promotes T Reg accumulation.

Metabolic reprogramming is a major driver of RCC pathogenesis and resistance to therapy. Key metabolic changes (particularly pronounced in ccRCC) include a significant Warburg effect, glutamine dependence, and the altered lipid and amino acid metabolism—resulting in high lactate production [57,96,101]. These changes result in a highly acidic TME (often pH < 6.5), wherein CA9-mediated acid regulation allows RCC cells to maintain viability under conditions that would be toxic to normal cells, providing a survival advantage during treatment [50,53,95]. This acidic extracellular compartment directly suppresses cytotoxic (CD8+) T-cell function and promotes the polarisation of TAMs to an M2 phenotype—a state known to be pro-neoplastic and immunosuppressive [112,113]. Moreover, studies have shown that defective antigen presentation by dendritic cells, T Reg expansion, and the enhanced expression of immune checkpoint molecules are cultivated in these conditions—collectively stifling effective physiologic immune surveillance as well as immune responses potentiated by immunotherapies [36,101,112].

### 2.4. Acid-Sensing G-Protein-Coupled Receptors in Cancer

Proton-sensing GPCRs represent a specialised class of pH sensors that translate extracellular acidification into intracellular signalling cascades. These receptors are activated by the protonation of histidine residues in their extracellular domains, with optimal activation occurring at pH values between 6.4 and 7.0. A family of proton-sensing GPCRs—namely GPR4, GPR65, and GPR68—has emerged as critical molecular sensors enabling cells to detect and respond to extracellular acidosis within the TME. These receptors are activated by extracellular protons, transducing acidosis-driven signals through multiple downstream G-protein signalling pathways to regulate a diverse range of cellular processes [65,80,81,82]. While these GPCRs exhibit context-dependent and cell-type-specific effects, growing evidence implicates their involvement in cancer progression through the modulation of proliferation, angiogenesis, invasion, metastasis, and, particularly, immune evasion [114,115]. Other GPCRs that can sense specific acidic molecules exist but are thought to play more specialist roles. GPR132, for example, functions as a lactate-sensing receptor, which responds to free lactate molecules and other organic acids produced by glycolytic metabolism. Coincidentally, it is also worth noting that GPR132 is overexpressed in ccRCC and demonstrates a negative association with survival [116].

The tissue-specific expression patterns of this family of proton-sensing GPCRs create opportunities for targeted therapeutic interventions. In cancer, proton-sensing GPCRs serve dual roles as both sensors of the acidic microenvironment and mediators of acid-adapted cellular responses. Tumour cell expression of these receptors often correlates with aggressive phenotypes and poor prognosis across multiple cancer types [81]. The upregulation of these receptors on cancer cells has been suggested as a potential drug target in various cancers—such as GPR68 in head and neck squamous cell cancers, wherein it mediates acid-induced cellular responses including proliferation, invasion, and drug resistance [117]. The exact role each receptor plays in carcinogenesis remains unclear; however, there is a growing base of evidence beginning to establish their relative presence, downstream signalling, and functional impact in different cancer types, as well as the druggability of these GPCRs as potential therapeutic targets [Table 1]. GPCRs on normal cells within the TME can, too, be coerced into facilitating cancer progression. GPR4, coupled predominantly to Gαs, has been shown to promote angiogenesis, endothelial cell permeability, and lymphatic metastasis in multiple solid tumours (including head and neck cancers and ovarian carcinoma), with its expression thought to contribute to tumour angiogenesis and immune evasion through endothelial cell activation and inflammatory mediator production [77].

### 2.5. GPR65 Antagonism as Cancer Therapy

**Figure 2:**
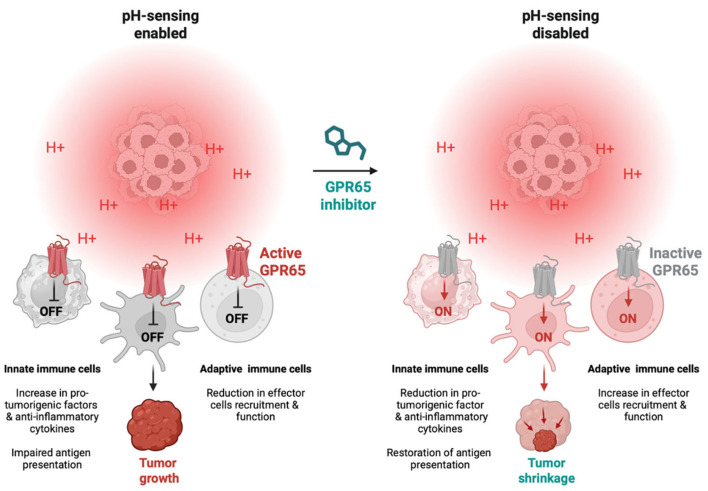
Proposed role for GPR65 in driving immune cell changes in the tumour microenvironment upon sensing acidic pHs. Acid-sensing on both innate and adaptive immune cells leading to immune suppression and tumour growth (**left**). Potential for GPR65 inhibitors to reverse this process, leading to tumour shrinkage (**right**). Created in https://Biorender.com.

Among the GPCRs, GPR65 has garnered particular attention as a promising immunotherapeutic target due to its predominant expression in immune cells and its central role in mediating acidosis-driven immunosuppression [64,66,67]. GPR65 couples to Gαs proteins and, upon activation by low extracellular pH, stimulates adenylate cyclase to increase intracellular cAMP production, leading to PKA activation and phosphorylation of the transcription factor CREB. This signalling cascade induces the expression of the inducible cAMP early repressor (ICER), producing profound shifts in macrophage and myeloid cell polarisation toward an immunosuppressive, pro-tumorigenic phenotype. These changes are characterised by elevated HMGB1 and IL-10 production, reduced pro-inflammatory cytokine secretion, and impaired antigen presentation [Figure 2] [59,65,71,80,81,123,130].

Mechanistic evidence from peer-reviewed studies implicates GPR65 as a modulator of cancer progression via the promotion of tumour cell adaptation to acidosis, conferring resultant survival benefits and immune evasion [64,65]. Functional studies demonstrate that GPR65 is often overexpressed in solid tumours—including RCC—and that its activation enhances tumour cell survival under acidic conditions by engaging the cAMP-PKA and ERK signalling pathways—supporting continued proliferation even in the hostile TME [59,60,63,65]. In experimental models, the overexpression of GPR65 in cancer cell lines (such as Lewis lung carcinoma) protected tumour cells from acidosis-induced death and stimulated in vivo tumour growth, while knockout or deficiency of GPR65 has been linked to diminished tumour progression in both solid and haematological cancers [59,60,66]. In the context of the immune microenvironment, GPR65 signalling has been demonstrated to skew macrophages and other immune cells toward immunosuppressive phenotypes, contributing to a TME that supports tumour progression [59,65,81]. This is further supported by a murine model that found that macrophage infiltration in obesity-associated colorectal and hepatocellular cancers promoted tumour growth, and that this was associated with elevated GPR65 expression in the TAMs of the subjects with obesity [61]. However, it is important to note that these observations are context-dependent; GPR65 knockout has also been associated with treatment resistance in haemato-oncology treatment resistance and increased secondary cancer [62,63]. Large-scale transcriptomic and immunohistochemical analyses show that GPR65 is significantly upregulated in RCC subtypes, particularly ccRCC, as well as other solid tumours relative to normal adjacent tissues [60,64]. Furthermore, given the high expression of CA9 in RCC tumours secondary to VHL loss and constitutive HIF activation, GPR65 represents a rational therapeutic target in this disease context [131]. Collectively, these findings support a mechanistic role for GPR65 in tumour survival, immune modulation, and progression within the context of RCC.

**Figure 3:**
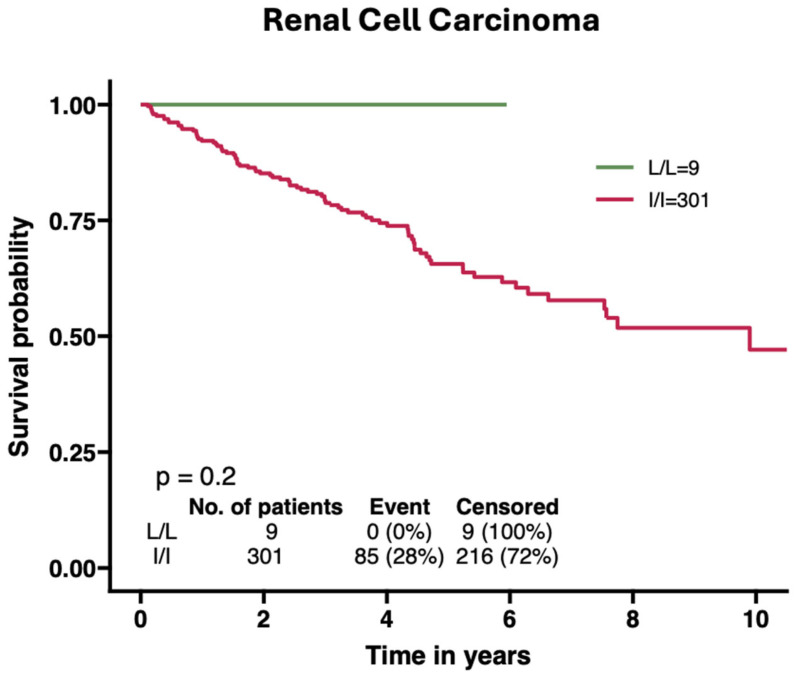
Kaplan–Meier curves with log rank test comparing RCC patients who are homozygous for the I231L SNP at GPR65 with patients that are wild-type at the same position in the TCGA.

Pathios Therapeutics Limited is a clinical-stage oncology biotech based in Oxford, UK. Pathios have developed and optimised a number of novel small-molecule agents that inhibit the proton-sensing ability of GPR65 by modulating downstream cAMP production—thus limiting the secondary messaging that normally takes place downstream of its activation within an acidic TME. Compelling human genetic validation for GPR65 as a therapeutic target comes from The Cancer Genome Atlas (TCGA) analyses performed by Pathios [Supplemental data], demonstrating that cancer patients homozygous for the hypomorphic I231L coding variant (rs3742704) (which exhibits significantly reduced GPR65 signalling capacity) display markedly improved overall survival across multiple solid tumour types compared to patients with wild-type GPR65 [Figure 3] [123]. This analysis was later independently replicated and published by Li et al., suggesting that GPR65 inhibition may confer a survival benefit in these solid tumour indications [65]. Preclinical studies have further substantiated this finding, showing that the genetic deletion of GPR65 in murine tumour models or pharmacological inhibition with selective small-molecule GPR65 antagonists developed by Pathios counteracts low-pH-induced immunosuppressive macrophage polarisation, restores anti-tumour immune activity, enhances infiltration of cytotoxic effector cells, and synergises with the anti-PD-1 checkpoint blockade to suppress tumour growth in syngeneic models [66,67,123]. In RCC specifically, ex vivo studies using tumour fragments from RCC patients have demonstrated that GPR65 inhibition modulates cytokine profiles within the TME, suggesting that the acid-sensing GPR65 pathway may contribute to the immunosuppressive landscape characteristic of this malignancy [65,67,123].

**Figure 4:**
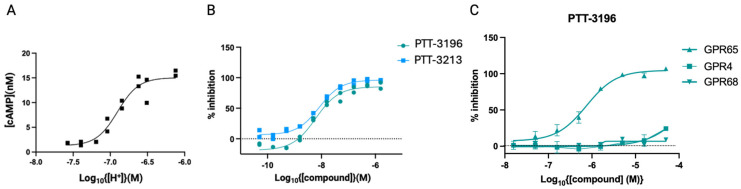
(**A**) cAMP generation following the exposure of human monocyte-derived macrophages (hMDMs) to a range of pHs. (**B**) Exemplary dose-dependent inhibition of cAMP by PTT-3196 and PTT-3213 in hMDMs exposed to a pH of 6.8. (**C**) Inhibition of cAMP by PTT-3196 in HEK cells induced to express GPR65, GPR4, or GPR68.

Tool molecules designed by Pathios to inhibit GPR65, PTT-3196, and PTT-3213 have been used to explore the effects of selective GPR65 inhibition in tumours. In macrophages, GPR65 signalling is responsible for an increased intracellular cAMP concentration [Figure 4A] in response to low-pH environments (Pathios proprietary data [Supplemental data], [92]). Using human monocyte-derived macrophages (hMDMs) generated from healthy volunteers, PTT-3196 and PTT-3213 were able to prevent GPR65 signalling and potently inhibit cAMP production at a pH of 6.8 [Figure 4B]. The molecules also demonstrated high selectivity for GPR65 compared to other members of the pH-sensing GPCR family—namely GPR4 and GPR68 [Figure 4C].

**Figure 5:**
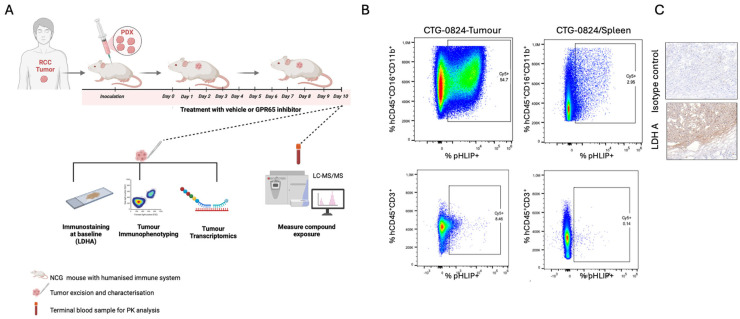
(**A**) Schematic of the RCC PDX CTG-0824 in vivo mouse model used to assess changes in immune markers in mice with a humanised immune system upon treatment with Pathio’s inhibitor PTT-3196. Tumour tissue was analysed at baseline to assess the expression of LDHA by IHC and by flow cytometry to detect the presence of low pH using the fluorescently labelled pHLIP peptide. A transcriptomics analysis of the tumours was performed in vehicle- vs. compound-treated mice at the end of the study (created in https://www.Biorender.com). (**B**) Immunophenotyping cytogram showing the presence of pHLIP + cells in the tumour and minimal signal in the spleen from the same PDX-tumour-bearing animal at baseline. (**C**) Representative image showing intense LDHA staining in CTG-0842 tumours relative to the isotype control at baseline.

In order to identify potential biomarkers of GPR65 inhibition and better understand the effects of this therapeutic approach in RCC, Pathios developed a novel murine model. This model utilises immunodeficient NOD/Prkdcem26Cd52Il2rgem26Cd22/NjuCrl (NCG) mice with RCC patient-derived xenograft (PDX) tumours (CTG-0842). The mice were reconstituted with a humanised immune system; CD34+ hematopoietic cells were engrafted, followed by the delivery of a plasmid encoding a cytokine cocktail (including GMCSF, IL3, IL4, and FLT3L) to expand the myeloid component, thereby recapitulating the macrophage-rich TME of human solid tumours [Figure 5A,B]. The RCC-derived tumours demonstrated high CA9 expression, and a high LDHA-to-LDHB expression ratio (>3:1) by transcriptomics. To confirm the presence of an acidic TME in the PDX tumours, a pH-low insertion peptide (pHLIP) was infused intravenously into the mice. This compound irreversibly binds to cells exposed to low-pH conditions with the degree of pHLIP staining—as assessed by flow cytometry—indicative of the amount of acidic environment exposure. Flow cytometry of cells extracted from PDX tumours showed pHLIP staining on both immune and non-immune cells within the tumour, including myeloid cells and T-cells [Figure 5B]. Conversely, only background staining was seen in cells extracted from the spleen [Figure 5C].

**Figure 6:**
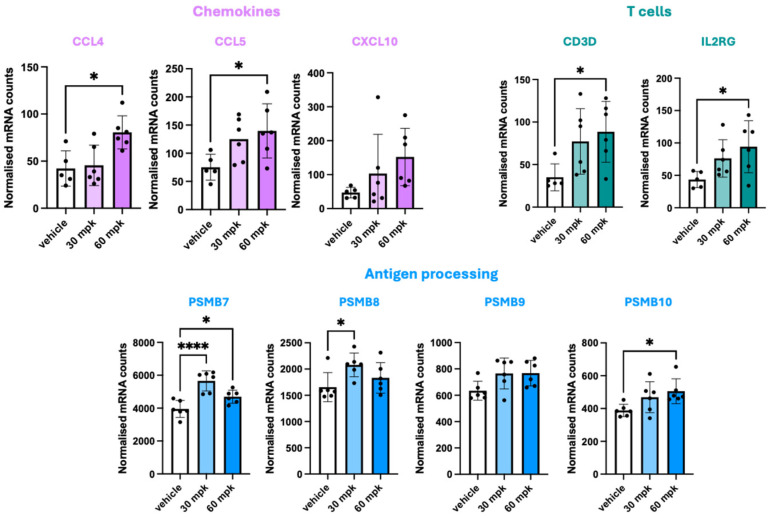
Gene expression changes measured by a NanoString Myeloid panel in tumours collected from CTG-0842-tumour-bearing mice at 2 h post-treatment. Normalised counts were compared from vehicle-treated vs. drug-treated mice using one-way ANOVA with Dunnett’s multiple comparison test (* *p* ≤ 0.05 and **** *p* ≤ 0.0001).

PTT-3196 is highly potent against human GPR65; however, it is significantly less potent on murine GPR65. To overcome the reduced potency in the mouse model, maximum tolerated doses were chosen based on a previous tolerability study in non-tumour-bearing mice that had undergone the same immune humanisation process. As a result, in the final CTG-0824 model, mice with established PDX tumours received 30 or 60 mg/kg of PTT-3196 orally, twice a day. The compound achieved a free concentration in the blood well above the known IC_50_ at the pH of 6.8 in the cAMP assay in macrophages (at least 170-fold). Gene expression changes measured in tumours from compound-treated versus vehicle-treated mice showed a statistically significant upregulation of genes related to chemokines involved in lymphocyte chemotaxis (CCL4, CCL5, and CXCL10), with a significant increase in CCL4 and CCL5 of 60 mg/kg (*p* < 0.05) [Figure 6]. Interestingly, this was mirrored by a significant increase in T-cell markers (CD3D and IL2RG) of 60 mg/kg, suggesting the recruitment of T-cells in the TME. PTT-3196 also led to an increase in the expression of PSMB7, PSMB8, PSMB9, and PSMB10—suggesting immunoproteasome induction, which is associated with improved MHC-I antigen processing and the enlargement of the repertoire of peptides displayed by tumour cells, and is linked to better responsiveness to T-cell-based immunotherapies [132,133,134]. Interestingly, T- and NK-cell chemoattractants (e.g., CCL3, CCL4, and CXCL10) were downregulated in human macrophages, whereas Th2-type cytokine genes were upregulated in human macrophages and NK cells (e.g., IL-10 and IL-13) exposed to low pH in vitro. Acidity also downregulated key effector cytokines (e.g., IL-2, IFNG, and TNFA) in human CD8 T-cells [Supplemental Figure S1]. These data from Pathios support the detrimental effect of a low pH on key innate and adaptive immune cells, with in vivo models with PTT-3196 suggesting that GPR65 inhibition reverses some of these immune-suppressive effects in the TME.

**Figure 7:**
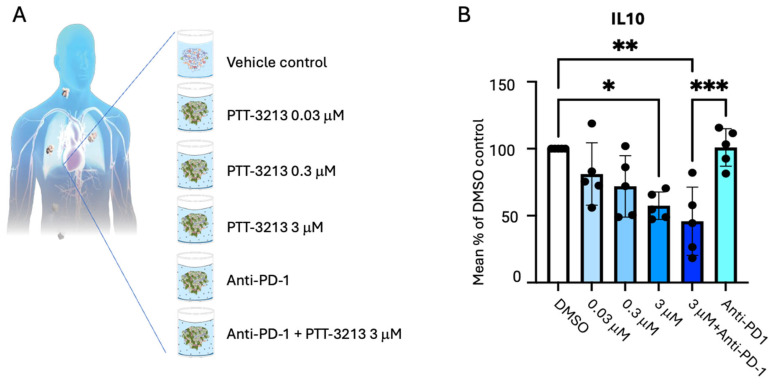
(**A**) Schematic of Pathios’ study conducted in human ccRCC tumour histocultures and treatment groups. (**B**) IL-10 was measured in supernatants collected at 24 and 48 h post-treatment for the total of n = 5 donors and is expressed as the mean percentage of control (DMSO, set at 100%). A one-way ANOVA with Tukey’s multiple comparison test was conducted (* *p* ≤ 0.05, ** *p* ≤ 0.01, and *** *p* ≤ 0.001). Data were combined from 24 and 48 h, depending on the donor, based on the max amount of IL-10 detected in the DMSO control.

To assess the effects of GPR65 inhibition in a fully human experimental system relevant to RCC, Pathios performed additional experiments using ccRCC histocultures derived from fresh tumour material donated by five patients. These tumour fragments could be maintained for 72 h in culture, suspended in proprietary medium containing autologous serum, without significant loss of cell viability (including naturally infiltrating immune cells). These histocultures were all shown to be highly positive for CA9 by immunostaining at baseline, a surrogate for tumour hypoxia and acidity (Supplementary Figure S2). The fragments were treated with PTT-3213 (at 0.03, 0.3, or 3 μM) alone, anti-PD-1 (132 μg/mL) alone, or PTT-3213 at 3 μM plus anti-PD-1 (132 μg/mL) for 72 h. Supernatants were then analysed to measure multiple cytokines and chemokines. Data showed that the most notable and consistent changes were observed in IL-10 production, which was significantly reduced (approximately 50%) by treatment with 3 μM PTT-3213 versus the vehicle [Figure 7A, Pathios proprietary data]. Interestingly, whilst treatment with anti-PD-1 did not elicit changes in IL-10, when PTT-3213 and anti-PD-1 were combined, there was a trend towards a stronger inhibition of IL-10 than that in 3 μM PTT-3213 alone [Figure 7B, Pathios proprietary data]. The reduction in IL-10 in this human system is, again, in line with data shared by Pathios in primary human macrophages stimulated with LPS and treated with PTT-3213 under low-pH conditions in vitro, whereby a low pH elicited a significant elevation in IL-10, which was prevented by GPR65 antagonists (Pathios proprietary data, [96]).

Following on from the preclinical data generated using PTT-3213 and PTT-3196 in RCC-derived tumour models, Pathios has taken an optimised, lead candidate of its GPR65 inhibitor into the clinic. PTT-4256, a first-in-class, oral, small-molecule GPR65 inhibitor, is currently under evaluation in the Phase I/II RAISIC-1 trial (Relief of Acidic Immune Suppression in Cancer; NCT06634849). This modular, multi-part, multi-arm, open-label study commenced patient dosing in November 2024. The initial module (Module A) is designed as a dose-escalation study employing a Bayesian optimal interval (BOIN) design to assess the safety, tolerability, pharmacokinetics, pharmacodynamics, and preliminary efficacy of PTT-4256 monotherapy in patients with advanced solid tumours, including melanoma, non-small-cell lung cancer, renal cell carcinoma, metastatic castrate-resistant prostate cancer, cervical cancer, triple-negative breast cancer, colorectal cancer, and gastric cancer, who have progressed on or are refractory to standard therapies. Subsequent modules are planned to explore the therapeutic potential of PTT-4256 in specific tumour types—starting with RCC—as a monotherapy, and in combination with established standards of care. There are no clinical or translational data available yet from this study [135].

At the time of writing, there are no other clinical-stage programmes targeting GPR65. However, a notable in vitro study validated a series of four novel GPR65 antagonists (SD2571, SD2593, SD2594, and SD2758) that were able enhance anti-tumour T-cell function and cytokine production in an acidic co-culture of immune cells and tumour cells. Using BTB095089 and ZINC13684400—commercially available allosteric agonists of GPR65—the pH-dependant antagonisim of GPR65 by these novel compounds was confirmed [65]. To date, these agents have not advanced into clinical study. While there are no other agents currently in clinical development specifically aimed at GPR65, there are studies exploring the therapeutic potential of investigational medicinal products targeting other components of the acidic TME—such as CA9 and MCT—in RCC [136,137,138,139,140,141,142,143,144]. However, these too are predominately in the early phases of development without mature clinical data [Table 2].

## 3. Conclusions and Future Directions

The treatment of RCC has undergone several major paradigm shifts over the past three decades, evolving from cytokine-based therapies, through VEGF-targeted TKIs, to the current era of precision immunotherapy. These advances have substantially improved outcomes, yet the challenge of durable disease control persists. Resistance to ICIs remains a major barrier, driven in part by the complex interplay of hypoxia, angiogenesis, and immune escape within the tumour microenvironment. Among these, tumour acidosis has emerged as a fundamental and underappreciated determinant of both disease biology and therapeutic resistance. Nevertheless, several hurdles remain before a full endorsement of this modality can be given. As discussed throughout this review, the majority of data available at present has been generated from in vitro models; given the complex nature of the TME—and the large cast of cellular players involved with GPR65 signalling—it is extremely unlikely that these models are able to meaningfully recapitulate in vivo TME conditions. Consideration must also be given to the promiscuity of the proton-sensing GPCR secondary messaging, as seen in animal models, and the likelihood of redundant acid-sensing pathways, raising questions about the durability of any benefit that may be derived from targeting this apparatus in isolation [88].

After dose-finding and expansion within clinical studies, robust human biomarker and safety data are needed to provide a more complete assessment of what, if any, significant intratumoral changes are induced by GPR65 blockade within RCC lesions. Given the relatively nascent mechanistic understanding of these pathways within the TME, there is a paucity of evidence exploring the role of GPCRs within this context in the literature. As a result, the authors of this review are sentient of the fact that a disproportionate amount of the primary data discussed in this review has come from a single, commercial source (i.e., Pathios)—with whom several co-authors are affiliated—introducing possible reporting bias. Moreover, some of these findings are preliminary and require independent validation. To limit this, references to previously presented data have been provided alongside Supplemental data from any unpublished, proprietary information from Pathios. The authors have also sought to provide the reader with a comprehensively referenced and critical overview of the biological rationale underpinning the thesis of review, to compensate for the limited availability of independent, peer-reviewed data on GPR65.

The acidic TME represents a fundamental alteration in cancer biology that drives disease progression, treatment resistance, and immune evasion through multiple interconnected mechanisms. The sophisticated pH-regulatory machinery involving carbonic anhydrases, sodium–hydrogen exchangers, and proton-sensing GPCRs creates a complex network that maintains the reversed pH gradient that is characteristic of solid tumours. In RCC, these mechanisms are particularly relevant given the near-ubiquitous CA9 overexpression in this solid tumour type and the emerging challenge of ICI treatment resistance in this indication [33,36,101]. The evidence discussed in this review highlights the role that the acidic TME plays in not only shaping tumour cell survival and metastatic potential, but also critically undermining anti-tumour immunity through impaired effector T-cell function and the polarisation of macrophages towards immunosuppressive phenotypes. In this context, acid-sensing GPCRs—and GPR65 in particular—provide a compelling mechanistic link between extracellular acidosis and immune dysfunction in RCC. At least in concept, antagonising proton-sensing could disrupt the immune–angiogenic loops that characterise RCC pathogenesis, thereby restoring effective immune surveillance and potentiating existing therapeutic strategies [31,101]. Preclinical studies support the ability of GPR65 antagonism to reverse acid-induced immune suppression and reprogramme the TME towards immune activation, while sparing normal pH homeostasis. Importantly, this strategy may hold promise across different treatment settings—as a monotherapy in heavily pre-treated patients, but also in combination with ICIs in earlier lines of therapy. In the latter case, GPR65 inhibition may also provide options for patients with primary or secondary refractory disease by aiding immune effector cell infiltration and persistence in the TME [36,56]. In RCC, particularly, GPR65 inhibition holds promise beyond its potential to bolster ICI activity—particularly given the impact of TME acidosis on PD-L1 expression via STAT pathways [46,47]. VEGF and HIFα are established therapeutic targets in RCC, with drugs such as sunitinib and belzutifan achieving monotherapy efficacy by exploiting this cancer’s dependence on angiogenesis [145,146]. Both VEGF signalling and HIF pathway activation are closely tied to the acidity–hypoxia axis observed in tumours [40,114]. Therefore, the inhibition of GPR65 in RCC offers the potential to concurrently disrupt both immune suppression and angiogenic pathways critical to tumour progression.

Although GPR65 blockade shows promise in mitigating acidosis-driven immune suppression within the TME, even from the limited evidence that exists, there are important limitations and context-dependent effects that warrant cautious interpretation. In various cancer types, GPR65 inhibition may inadvertently promote pro-tumour phenotypes—either by driving compensatory metabolic reprogramming or by altering macrophage polarisation in a way that supports tumour growth under specific conditions. Some studies report that a loss or reduction in GPR65 activity can foster resistance to apoptosis, facilitate immune escape, and even increase the risk of secondary cancers [62,63,71]. These paradoxical effects may be highly tumour- and TME-phenotype-specific, due to the multiple roles GPR65 signalling plays across different immune and non-immune cell compartments [60,65,147]. For RCC, additional challenges may include redundant metabolic pathways and spatial heterogeneity within the TME. GPR65 blockade may not uniformly remodel immune landscapes across all tumour regions and could inadvertently support cancer stem cell niches or create selection pressures that confer survival advantages to more aggressive cancer cells [97,99]. Furthermore, RCC’s strong reliance on CA9 and MCT networks may limit the monotherapy efficacy of GPR65-targeted strategies; signal ‘noise’ induced downstream of a GPR65 blockade between immune and metabolic signalling pathways could blunt therapeutic efficacy or induce unforeseen resistance mechanisms [99,109,110,147].

Despite these challenges, the development of first-in-class GPR65 inhibitors—such as PTT-4256—marks a significant milestone. By directly addressing an underexplored axis of tumour immunology, PTT-4256 and similar agents offer the prospect of reprogramming the TME to overcome treatment resistance—one of the most pressing unmet needs in advanced RCC. Emerging clinical data from the early-phase RAISIC-1 trial will be pivotal in clarifying safety, tolerability, and preliminary efficacy, and will guide the rational design of future combination approaches. Formal mechanistic studies in RCC models—as well as other solid tumour indications—are required to more fully define the precise cellular circuits engaged by GPR65, as well as their interplay with angiogenic and metabolic pathways. Parallel efforts should focus on biomarker mapping of acid-sensing pathways in clinical samples to establish predictive markers of response and to stratify patients most likely to benefit from this therapeutic strategy. Beyond tissue-based clinical sampling, biomarker-led approaches to targeting acid-sensing apparatus may be aided by the increasingly sophisticated non-invasive techniques of intratumoral pH measurement. New imaging techniques such as chemical exchange saturation transfer (CEST) MRI and photoacoustic imaging using pH-responsive nanoparticles have demonstrated the ability to measure TME pH in vivo with high spatial resolution [148,149,150]. While these methods have shown promise for improving diagnosis in preclinical models, they may also have utility in guiding therapies targeted at the acidic TME.

RCC has benefitted from the advent of immunotherapy, arguably more so than many other solid tumours. Yet, significant challenges remain in extending the benefits of immunotherapy to all patients. Targeting acid-sensing pathways represents a bold and scientifically grounded strategy to dismantle one of the key barriers to durable immune control. The integration of molecular, immunologic, and microenvironmental therapies may define the next phase of RCC treatment—with GPR65 inhibition having the potential to play a central role in this multimodal approach.

## Figures and Tables

**Table 1:** Comparative overview of proton-sensing GPCRs across major cancer types: expression patterns, signalling mechanisms, functional implications, and druggability.

Receptor	Cancer Type	Expression Pattern	G-Protein Coupling	Phenotype/Functional Impact	Druggability Status	References
*GPR4*	Colorectal cancer	Upregulated	Gαs, Gαq/11	Promotes proliferation and metastasis via the Hippo/YAP1 pathway	Small-molecule antagonist NE 52-QQ57 reduces lymphangiogenesis and lymph node (LN) metastasis	1. The Roles of Proton-Sensing G-Protein-Coupled Receptors in Inflammation and Cancer. Frontiers in Endocrinology [77]. 2. Proton-sensing ion channels, GPCRs and calcium signaling regulated by them. Frontiers in Cell and Developmental Biology [118]. 3. Proton Sensing GPCRs: The missing link to Warburg’s cancer cell metabolism. Frontiers in Physiology [119]. 4. Increased proton-sensing receptor GPR4 signalling promotes colorectal cancer progression [120]. 5. Loss of proton-sensing GPR4 reduces tumor progression and metastasis. Cancer Science [121]. 6. The Proton-Sensing G-Protein Coupled Receptor GPR4 Promotes Angiogenesis in Head and Neck Cancer. PLOS ONE [122]. 7. Acidic tumor microenvironment and pH-sensing G protein-coupled receptors. Frontiers in Physiology [115]. 8. Expression profiles of proton-sensing G-protein coupled receptors in melanoma. Scientific Reports [81]. 9. GPR65 as a potential immune checkpoint regulates the immune microenvironment. Heliyon [64]. 10. Proton-sensing G protein-coupled receptors. Pharmacological Reviews [114].
	Ovarian cancer	Upregulated; correlates with microvascular density	Gαs	Promotes angiogenesis and tumour growth	Preclinical antagonists	
	Head and neck cancer	Upregulated in tumour endothelium	Gαs	Promotes angiogenesis; secretion of IL-6, IL-8, and VEGF	GPR4 deficiency reduces tumour angiogenesis	
	Melanoma	Context-dependent (high in dermal melanoma)	Gαs	Dual role: inhibits migration (overexpression) or promotes migration (pH 6.5–7.5)	GPR4 knockout reduces metastasis	
	Breast cancer	Upregulated	Gαs	Promotes tumour growth and angiogenesis	GPR4-KO reduces tumour growth	
	Renal cell carcinoma	Detected	Gαs	Role unclear; likely involved in acidosis sensing	No specific inhibitors reported	
*GPR65 (TDAG8)*	Pan-cancer analysis	Variable (notable elevations in glioblastoma, renal cell carcinoma [clear-cell and papillary subtypes] and melanoma)	Gαs, Gα13 (context-dependent)	Human genetic validation: I231L variant (reduced signalling) associated with improved survival across multiple solid tumours	PTT-4256: first-in-class inhibitor; RAISIC-1 Phase I/II trial (NCT06634849) ongoing in solid tumours including RCC	1. Ludwig MG et al. (2013). Acidic tumor microenvironment and pH-sensing G protein-coupled receptors. Frontiers in Physiology [115]. 2. The Roles of Proton-Sensing G-Protein-Coupled Receptors in Inflammation and Cancer. Frontiers in Endocrinology [77]. 3. In silico and pharmacological evaluation of GPR65 as a cancer immuno-oncology target. Frontiers in Immunology [65]. 4. Chen X et al. (2024). GPR65 contributes to constructing immunosuppressive tumor microenvironment. Journal of Translational Medicine [59]. 5. GPR65 as a potential immune checkpoint regulates the immune microenvironment. Heliyon [64]. 6. Zhang Y et al. (2025). GPR65 is a novel immune biomarker and regulates the immune microenvironment. Scientific Reports [60]. 7. The acid-sensing receptor GPR65 on tumor macrophages drives immunosuppression [61]. 8. Abstract 668: The translational biology of small molecule GPR65 inhibitors [123]. 9. Pathios Therapeutics Announces Dosing of First Patient in Phase 1/2 Clinical Study of GPR65 Inhibitor, PTT-4256 [124]. 10. An Open Label Dose Finding Study of PTT-4256 in Patients With Solid Tumours (RAISIC-1) [125]. 11. Expression profiles of proton-sensing G-protein coupled receptors in melanoma. Scientific Reports [81]. 12. Acidosis-mediated increase in IFN-γ-induced PD-L1 expression on cancer cells as an immune escape mechanism in solid tumors. OncoImmunology [47].
	Melanoma	High (epidermal); weak (dermal)	Gαs	Promotes survival and proliferation	PTT-3213 (preclinical)	
	Renal cell carcinoma	Elevated in clear-cell subtype	Gαs	Mediates immunosuppression via macrophage polarisation in acidic TMEs	PTT-4256 in Phase I/II clinical trial (RAISIC-1)	
	Lymphoma	Decreased >50% vs. normal lymphoid tissue	Gαs	Promotes apoptosis; inhibits c-Myc expression (tumour-suppressor role)	No inhibitors reported	
	Non-small-cell lung cancer	Detected	Gαs	Promotes survival under acidic conditions	No specific inhibitors for NSCLC	
	Head and neck cancer	Detected; correlates with PD-L1 expression	Gαs	Induces PD-L1 upregulation; promotes immune evasion	GPR65 inhibition synergises with anti-PD-1	
	Colon cancer	Detected	Gαs	Variable	PTT-4256 clinical trial	
	Breast cancer	Detected	Gαs	Variable	PTT-4256 clinical trial	
*GPR68 (OGR1)*	Pancreatic cancer	10.5-fold higher vs. healthy controls	Gαq/11, MAPK	Context-dependent; promotes tumorigenesis, growth, and metastasis	Small-molecule inhibitor shows efficacy in IBD models; challenging druggability for cancer	1. GPR68: An Emerging Drug Target in Cancer. International Journal of Molecular Sciences [117]. 2. GPR68-ATF4 signaling is a novel prosurvival pathway in glioblastoma [126]. 3. Proton-sensing G protein-coupled receptors as regulators of cell proliferation and migration. International Journal of Molecular Sciences [114]. 4. Mechanisms and therapeutic promise of GPR68 (OGR1) in aging and cancer. Frontiers in Aging [127]. 5. Patent US20250099438A1 (2024). Small molecule inhibitors of GPCR GPR68 and related methods. Google Patents [128]. 6. Acidic tumor microenvironment and pH-sensing G protein-coupled receptors. Frontiers in Physiology [115]. 7. Gu Q et al. (2021). Inhibition of host Ogr1 enhances effector CD8+ T-cell function. Cancer Gene Therapy [129]. 8. Proton-sensing G protein-coupled receptors. Pharmacological Reviews [114].
	Glioblastoma	Upregulated	Gαq	Pro-tumorigenic via ATF4 signalling; promotes proliferation	Positive allosteric modulators developed	
	Prostate cancer	Lower in metastatic lesions vs. primary tumour	Gαq	Tumour suppressor: reduces metastasis when overexpressed	OGR1 inhibition enhances CD8+ T-cell function (preclinical)	
	Colorectal cancer	High in cancer-associated fibroblasts (CAFs) and tumour cells	Gαq	Context-dependent	Preclinical inhibitors tested	
	Melanoma	Upregulated	Gαq	Context-dependent	No specific inhibitors reported	
	Head and neck cancer	Significantly higher vs. normal	Gαq	Promotes tumour progression	No cancer-specific inhibitors in clinical development	
	Breast cancer	High in CAFs	Gαq	Variable	Preclinical only	

**Table 2:** Table summarising investigational medicinal agents targeting CA9 or MCT currently in preclinical or early clinical development for RCC.

Agent	Mechanism	Clinical Phase/Setting	Positive Findings	Limitations	References
Girentuximab (anti-CA9 mAb)	CA9 antibody (imaging, therapy)	Phase III (ARISER); PET imaging	Effective for [^89^Zr]Zr-girentuximab PET-based diagnostics in ccRCC imaging; some disease control as therapy with potential for synergistic use in combination with HIF2α inhibitors or TKIs	No disease-free/overall survival (OS) benefit in adjuvant RCC; limited as therapeutic monotherapy	[136,137,140]
CA9hu-1 (humanised antibody)	CA9 antibody (immunotherapy)	Phase I (metastatic/refractory RCC)	Strong ADCC, CDC, and CAIX binding in preclinical and early human studies	No efficacy data from large patient cohorts published	[138,139]
AZD3965 (MCT1 inhibitor)	MCT1 blockade	Phase I (advanced cancers incl. RCC)	Target engagement and anti-tumour effects demonstrated in preclinical RCC Xenograft data	Resistance in MCT4^high^ tumours; Phase I outcomes for RCC group not specifically published	[141,142]
Syrosingopine (MCT1/MCT4 inhibitor)	MCT1 and MCT4 blockade	Preclinical/in vivo studies only	Reduced lactate/acidification, proliferation; increased apoptosis in vitro, especially in combination settings	No tumour regression or survival benefit seen with in vivo xenograft RCC models	[143,144]

## Data Availability

The original contributions presented in this study are included in the article/Supplementary Material. Further inquiries can be directed to the corresponding author. References [151,152,153,154,155,156,157,158,159,160,161,162,163,164] are cited in the Supplementary Materials’ Methods section.

## Supplementary Materials

The following supporting information can be downloaded at https://www.mdpi.com/article/10.3390/cancers17233883/s1, Figure S1: gene expression changes measured by NanoString nCounter after four hours-exposure to pH 6.8 (human CD8 T cells and human monocyte-derived macrophages (MDMs)) or pH 6.5 (NK cells) vs pH 7.4 in vitro; Figure S2: A: Table with information on patients’ ccRCC samples used in the tumour histocultures study with PTT-3213. B: representative image of CA9 staining from ccRCC histocultures at baseline on Formalin Fixed paraffine embedded tissue. C: %CA9 staining in all tumour histocultures at baseline.
