# Correction: Grant et al. Low pH, High Stakes: A Narrative Review Exploring the Acid-Sensing GPR65 Pathway as a Novel Approach in Renal Cell Carcinoma. *Cancers* 2025, *17*, 3883

**DOI:** 10.3390/cancers18050760

**Published:** 2026-02-27

**Authors:** Michael Grant, Barbara Cipriani, Alastair Corbin, David Miller, Alan Naylor, Stuart Hughes, Tom McCarthy, Sumeet Ambarkhane, Danish Memon, Michael Millward, Sumanta Pal, Ignacio Melero

**Affiliations:** 1 St Bartholomew’s Hospital, London EC1A 7BE, UK; 2 Weatherden Limited, London WC1V 6DF, UK; 3 Pathios Therapeutics Limited, Oxford OX2 6HJ, UK; barbara@pathios.com (B.C.); david@pathios.com (D.M.); alan@pathios.com (A.N.); stuart.hughes@pathios.com (S.H.); tom@pathios.com (T.M.); sumeet.ambarkhane@pathios.com (S.A.); 4 Molecule to Medicine, Oxford OX1 4PS, UK; danish@m2m.bio; 5 School of Medicine, University of Western Australia and Linear Clinical Research, Perth, WA 6009, Australia; michael.millward@uwa.edu.au; 6 City of Hope Comprehensive Cancer Center, City of Hope, Duarte, CA 91010, USA; spal@coh.org; 7 Department of Immunology and Immunotherapy, Clinica Universidad de Navarra, 31008 Pamplona, Spain; imelero@unav.es

**Figure/Legend**

In the original publication [1], there was a mistake in Figure 6 and its legend: letter headings were not needed. Please use the amended Figure 6 with its legend below. The authors state that the scientific conclusions are unaffected. This correction was approved by the Academic Editor. The original publication has also been updated.

## Figures and Tables

**Figure 6:**
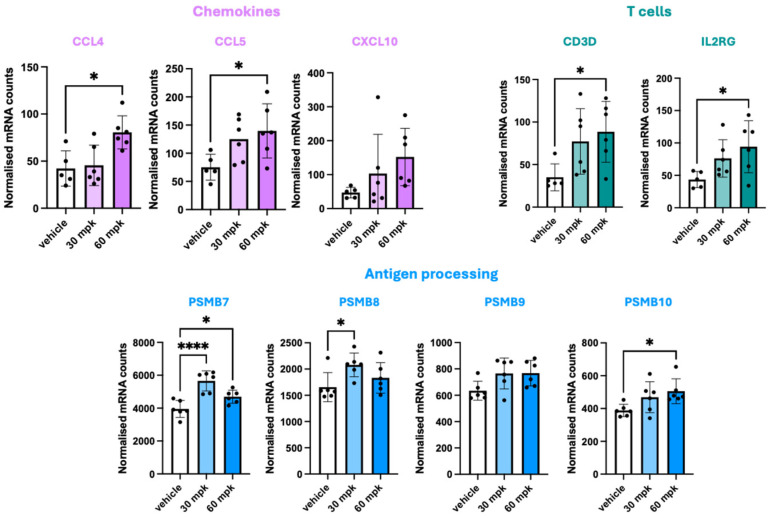
Gene expression changes measured by a NanoString Myeloid panel in tumours collected from CTG-0842-tumour-bearing mice at 2 h post-treatment. Normalised counts were compared from vehicle-treated vs. drug-treated mice using one-way ANOVA with Dunnett’s multiple comparison test (* *p* ≤ 0.05 and **** *p* ≤ 0.0001).

## References

[1] Grant M., Cipriani B., Corbin A., Miller D., Naylor A., Hughes S., McCarthy T., Ambarkhane S., Memon D., Millward M. (2025). Low pH, High Stakes: A Narrative Review Exploring the Acid-Sensing GPR65 Pathway as a Novel Approach in Renal Cell Carcinoma. Cancers.

