# Supplementary material for: Low pH, High Stakes: A Narrative Review Exploring the Acid-Sensing GPR65 Pathway as a Novel Approach in Renal Cell Carcinoma
Source: Cancers (Basel). 2025 Dec 4;17(23):3883. doi: 10.3390/cancers17233883 (PMC12691181; doi:10.3390/cancers17233883)
Supplement: Supplementary file 1 [file cancers-17-03883-s001.zip › cancers-3986285-supplementary/cancers-3986285/cancers-3986285-supplementary.pdf]

Supplementary Figures and Methods

Figures

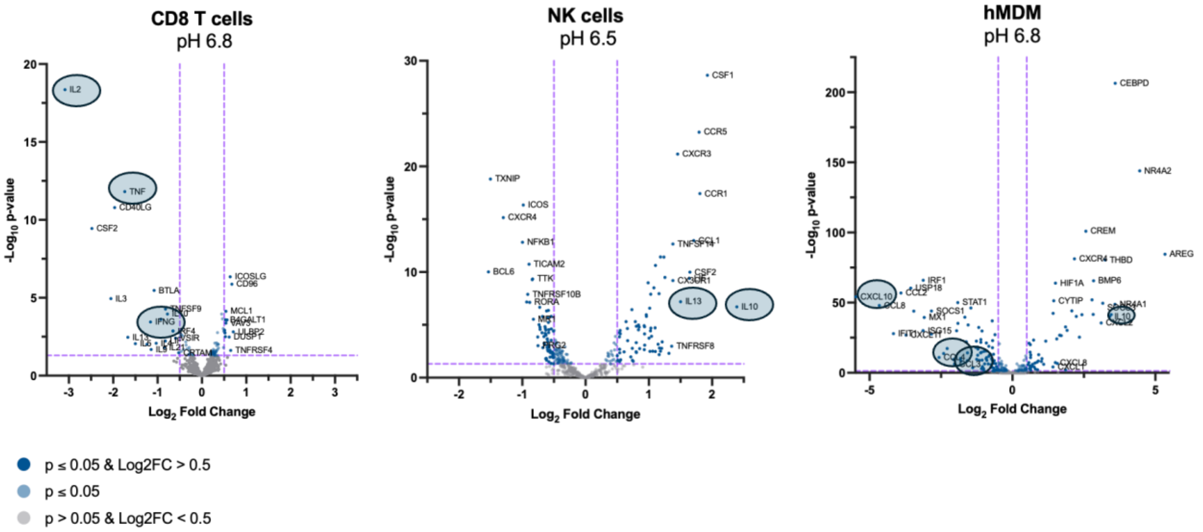

Supplementary Figure S1. gene expression changes measured by NanoString nCounter after four hours-exposure to pH 6.8 ((human CD8 T cells and human monocyte-derived macrophages (MDMs)) or pH 6.5 (NK cells) vs pH 7.4 in vitro.

A

No.	ID	Age	Gender	Grade	Stage
1	FBB1F220067	50	Male	IV	II
2	FBB1F220075	45	Female	I	I
3	FBB1F220081	69	Male	I	I
4	FBB1F220092	65	Male	I	II
5	FBB1F2200136	53	Male	I	I

B

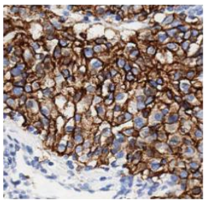

C

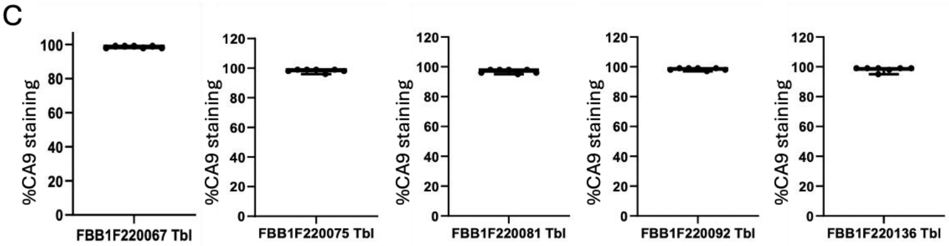

Supplementary Figure S2. A: Table with information on patients' ccRCC samples used in the tumour histocultures study with PTT-3213. B: representative image of CA9 staining from ccRCC histocultures at baseline on Formalin Fixed paraffine embedded tissue. C: %CA9 staining in all tumour histocultures at baseline.

Methods

TCGA survival analysis according to genotype

Germline variant calls (rs3742704) of GPR65 were obtained for TCGA patients from dbGAP (phs000178.v11.p8). Survival data of clear cell renal cell carcinoma patients (TCGA KIRC) was downloaded from Liu et al, 2018 to compare outcomes of 310 patients stratified based on rs3742704 SNP status (1). Patients with heterozygous rs3742704 SNP status were excluded from the analysis.

Kaplan-Meier analysis was performed using survfit() function in survival (v3.6-4) package in R (v4.3.3). A log-rank test was used to compute p value to compare overall survival of patients with wild type and homozygous hypermorphic SNP using survdiff() function also part of survival package in R. Survival curves were visualised using ggsurvplot() function in survminer (v0.4.9) package in R.

In vivo RCC PDX and characterisation

Immunodeficient NCG mice (Charles River) were engrafted intravenously with cord blood-derived CD34+ hematopoietic stem cells two days after chemical myeloablative treatment with Busulphan. The CD34+ cord blood cells were derived from multiple donors and randomized across the groups. Fourteen weeks after cell injection, engraftment level was monitored by analysing the percentage of human CD45+ cells among total blood leukocytes by flow cytometry. Only mice with a humanization rate (hCD45/Total CD45) above 25% were recruited into the study. Mice were inoculated with fragments of CTG-084 renal cell carcinoma tumours (previously expanded in stock animals) unilaterally on the left flank. The animals were injected IV with a plasmid encoding myeloid-boosting factors (GMCSF, IL3, IL4, FLT3L) 1-week after tumour fragment implantation. Tumour growth monitored and when tumours reached an average tumour volume of 150-300 mm^3 animals were randomised into treatment groups (with PTT-3196 at 30 or 60 mg/kg dissolved in 5% DMSO, 5% Kolliphor HS15 and 90% water) or with vehicle and dosing initiated on Day 0. Compound or vehicle administration was carried out BID with a 10-hours interval for 5 days/week and 2 days OFF (weekend break). In total twelve animals were treated, six per group, when tumours reached a volume of 500-750 mm^3.· Terminal whole blood samples were collected 2 hours post final dose to measure compound concentration. Blood was collected into LitHep-coated tubes (Sarstedt Microvette CB300 LH) and placed on wet ice for up to 30 minutes. A 40µl aliquot of blood was mixed with a 40µl aliquot of deionised water and gently mixed. A 70 µl aliquot of 1:1blood:water was then placed into a 96 well MT plate (PP V-bottom Greiner Bio-One 651201) on dry ice and then transferred to a freezer at approx. -80 ^oC overnight until analysed by LC-MS. For LC-MS duplicate compound DMSO stock solutions were serially diluted with DMSO to generate calibration standard and QC spiking solutions. Matrix-matched calibration standard and QC Samples were prepared by fortifying 95 µL of blank matrix with 5 µL of DMSO spiking solution. A 20 µL aliquot of each calibration standard, QC sample and Test Sample was subsampled. Additional 20 µL aliquots of blank matrix were also sampled. All Test Samples, Calibration Standards, QC Samples and Blank Matrix samples were quenched in 80 µL Acetonitrile containing internal standard, mixed (500 rpm, 1 min) then centrifuged at

3500 rpm for 10 minutes at 4°C. An aliquot of the resultant supernatant was then transferred to an analytical plate, with water added, prior to analysis by LC-MS/MS. Tumour tissue was collected to study gene expression using the Human Pan Cancer Immune Profiling nCounter panel by NanoString, which includes 770 genes relevant to cancer biology and anti-tumour immune responses. In a previous pilot study the PDX CTG-0824 was shown to display an acidic tumour microenvironment after implantation and growth in NCG mice demonstrated by positive immunostaining of tumour and tumour-infiltrating immune cells with the a Cy5-conjugated pHLIP peptide (variant 3, with sequence A[C-Cy5]DDQNPWRAYLDLLFPTDTLLLDLLW, by Pepceuticals, now part of Biosynth) after this was injected IV in 100ml at 40mM in PBS 24 hours prior to collecting tumours and spleens (as control tissue) and processing via flow cytometry. Antibodies used for immunophenotyping were: BV421 CD163 (Biolegend), BV510 CD45, FVS575V Viability dye, BV711 CD16, BV786 CD3 (Beckton Dickinson), Viobright FITC CD86, PE CD14 , PE-Vio770 CD11b , APC Vio770 mCD45 (Miltenyi).
LDHA immunohistochemistry was performed on CTG-0824 PDX cryosections (6mm). Sections were placed onto superfrost slides, dried in the cryostat chamber for fifteen minutes before -80°C storage.
Immunostaining was achieved with LDHA (C4B5) Rabbit mAb #3582 (Cell Signaling Technology) or with the Isotype Control Ab172730 (Abcam) by incubating overnight at 4°C followed by the Anti-Rabbit HRP-DAB Cell & Tissue Staining Kit . Finally, cell nuclei were counterstained with Haematoxylin and slide cover mounted.

Human immune cell transcriptomics analysis after in vitro pH treatment

Human PBMCs from healthy donors (n=6 in each study, data from two studies provided) were prepared from buffy coats. Monocytes were isolated using immuno-magnetic separation and differentiated into M2-like macrophages over eight days in the presence of human recombinant M-CSF (50 ng/mL) in culture medium RPMI-10 (RPMI-1640 containing 10 % FBS, 100 U/mL penicillin, 100 µg/mL streptomycin, 2 mM L-glutamine and 50 µM 2-Mercaptoethanol). Additional medium containing M-CSF was added on day three and then replenished on day six. Cells were plated on UpCell® dishes to allow for non-enzymatic collection at the end of culture. Following differentiation, on day 8, cells were detached and re-suspended in culture medium at 1 x 10^6 cells/mL, and 0.5 x 10^6 cells (500 µL) were seeded per well of a regular or UpCellTM 48-well flat-bottomed plate. On day 9, medium from pre-plated macrophages was removed and cells from each donor were treated with serum-free medium at either pH 7.4 or 6.8.
Human CD8+T cells were isolated from PBMC (n=6 donors) via negative selection and activated with anti-CD3/anti-CD28 coated Dynabeads™ for 4 hours at pH 7.4 or 6.8. At the end of the 4 hours Dynabeads were removed by magnetic isolation and cells collected. NK cells were obtained by expanding them from PBMCs cultured for 13 days using the NK MACS medium (Miltenyi) supplemented with recombinant human IL-2 (500IU/mL) and IL-15 (140 IU/mL) as outlined in Miltenyi protocol. Cells were then washed and plated at pH 7.4 or 6.8 for 4 hours. After collection of all immune cells RNA was extracted using the RNeasy 96 kit (Qiagen).After RNA QC 100 ng of RNA per sample was submitted for gene expression analysis using the Nanostring nCounter human myeloid panel for macrophages, the Human Exhaustion panel for T cells and Cancer

Human immune profiling panel for NK cells. Gene expression changes expressed as Log2 Fold Change (Log2FC) between the neutral pH control condition (pH 7.4) and the acidic condition (pH 6.8) were generated in R and displayed in the form of volcano plots.

Patient-derived RCC tumour histocultures

ccRCC tissues were processed to generate sub-500 mm thick sections using a vibratome. Sections were distributed into 7 arms (or wells). One arm was submitted for pre-culture histopathological analysis. The remaining six arms were cultured in medium supplemented with autologous plasma and FBS. The arms were cultured either with vehicle i.e., DMSO, or treated with PTT 3213 or Anti-PD-1 alone and in combination as shown in Figure 4 and cultured for 72 hours. PrestoBlue HS reagent (Thermo Scientific) assay was performed pre and post culture to assess viability. Culture supernatant was collected from all arms pre-treatment (TBL) and at 24-hour intervals until termination followed by replenishing with fresh media along with treatment agents.
The collected supernatants were stored at -80° C for cytokine analysis.
Immunostaining: to detect Carbonic Anhydrase 9 (CA9) at baseline the rabbit monoclonal antibody EPR4151 (Abcam, 1:200 dilution) and Ventana benchmark XT (BenchMark ULTRAIHC/ISH System, Roche) were used and the detection was done using ultraView universal DAB detection Ventana kit (Roche). Procartaplex 20 marker panel (Cat: PPX-20-MXEPUKN) was used to measure cytokines.

Evidence for highlighting selected biomarkers in reported data

- **CCL4 and CCL5**

  These have been shown to play a role in attracting adaptive immune cells as well as cDC1 cells to the tumour microenvironment to promote anti-tumour immunity. However, Tregs and TAMs recruitment are also induced via the CCL4/CCL5/CCR5 axis. This review article highlights the role of various chemokine and chemokine receptors in cancer (2). CCL4 and CCL5 can also be expressed by tumour cells and in the context of ccRCC this is associated with spatial association with PD-1+ T cells (3).

  In the context of this work, being that GPR65 is expressed mainly in immune cells, we would expect that the most prominent effect of GPR65 inhibition would be to elicit changes in the production of these chemokines in the immune cells infiltrating tumours, which would maintain effector status and could lead to attracting more immune cells into the TME.

- **CD3D**

  This is pan-T cell marker, required for the development and function of T cells (4,5). This protein is essential for the expression of the abTCR in T cells (6).The CD3 delta chain was also shown to mediate the association of CD8 with the TCR and for the positive selection and activation of CD8+ T cells (7).

- **IL2RG**

It is the common chain for several cytokine receptors such as IL-2, IL-4, IL-7, IL-9, IL-15, IL-21 (8). Mutations in the IL2RG gene cause X-linked severe combined immunodeficiency (X-SCID), a severe disorder characterized by a lack of functional T cells suggesting a pivotal role in immunity (9).

PSMB7,8,9,10 These are components of the proteasome and immunoproteasome (IP), crucial for antigen presentation. They are expressed in antigen presenting cells and can be induced in tumour cells by an inflammatory tumour microenvironment (10). There is evidence that expression of PSMB8 is correlated with better prognosis in breast cancer and that PSMB8 and 9 are correlated with better prognosis in melanoma (11) In general, IP component expression is correlated with better survival in certain malignancies such as skin cutaneous melanoma (SKCM), cervical squamous cell carcinoma (CESC), breast invasive carcinoma (BRCA), bladder urothelial carcinoma (BLCA), mesothelioma (MESO) and sarcoma (SARC) whereas is correlated with poor survival in kidney renal clear cell carcinoma (KIRC), acute myeloid leukaemia (LAML), brain lower grade glioma (LGG) and uveal melanoma (UVM) (12).

- **IL-10**
  Historically considered a potent pro-tumorigenic cytokine. High levels of baseline serum IL-10 are associated with reduced clinical benefit from first-line immune checkpoint inhibitor therapy in advanced renal cell carcinoma (13). However, more recent studies suggest it may play a role in anti-tumour immunity as well. The role of IL-10 therefore appears to be complex and context dependent. For example it is pro-tumorigenic on tumour cells but anti-tumorigenic on immune cells. Targeting IL-10 has not yet been validated in the clinic for cancer (14).

Acknowledgements:

Pathios would like to acknowledge Sygnature Discovery and Concept Life Sciences for running in vitro studies, Champs Oncology for performing the RCC PDX model, ImaBiotech(now Aliribio) for IHC of the PDX tumours, Farcast Bioscience for the patient-derived ex vivo histoculture study an Alison Munroe at HTPU Microarray Services at The University of Edinburgh for running NanoString gene expression studies.

References:

1. Liu J, Lichtenberg T, Hoadley KA, Poisson LM, Lazar AJ, Cherniack AD, et al. An Integrated TCGA Pan-Cancer Clinical Data Resource to Drive High-Quality Survival Outcome Analytics. Cell [Internet]. 2018 Apr 5 [cited 2025 Nov 16];173(2):400-416.e11. Available from: https://pubmed.ncbi.nlm.nih.gov/29625055/

2. Ozga AJ, Chow MT, Luster AD. Chemokines and the immune response to cancer. Immunity [Internet]. 2021 May 11 [cited 2025 Nov 16];54(5):859–74. Available from: https://www.sciencedirect.com/science/article/pii/S1074761321000352

3. Miheecheva N, Postovalova E, Lyu Y, Ramachandran A, Bagaev A, Svekolkin V, et al. Multiregional single-cell proteogenomic analysis of ccRCC reveals cytokine drivers of intratumor spatial heterogeneity. Cell Rep [Internet]. 2022 Aug 16 [cited 2025 Nov 16];40(7):111180. Available from: https://www.sciencedirect.com/science/article/pii/S2211124722009937#sec3

4. Basile G de Saint, Geissmann F, Flori E, Uring-Lambert B, Soudais C, Cavazzana-Calvo M, et al. Severe combined immunodeficiency caused by deficiency in either the delta or the epsilon subunit of CD3. J Clin Invest [Internet]. 2004 Nov 15 [cited 2025 Nov 16];114(10):1512–7. Available from: https://pubmed.ncbi.nlm.nih.gov/15546002/

5. Roifman CM. CD3 delta immunodeficiency. Curr Opin Allergy Clin Immunol [Internet]. 2004 Dec [cited 2025 Nov 16];4(6):479–84. Available from: https://pubmed.ncbi.nlm.nih.gov/15640687/

6. Buferne M, Luton F, Letourneur F, Hoeveler A, Couez D, Barad M, et al. Role of CD3 delta in surface expression of the TCR/CD3 complex and in activation for killing analyzed with a CD3 delta-negative cytotoxic T lymphocyte variant. The Journal of Immunology [Internet]. 1992 Feb 1 [cited 2025 Nov 16];148(3):657–64. Available from: https://dx.doi.org/10.4049/jimmunol.148.3.657

7. Doucey MA, Goffin L, Naeher D, Michielin O, Baumgärtner P, Guillaume P, et al. CD3δ establishes a functional link between the T cell receptor and CD8. Journal of Biological Chemistry [Internet]. 2003 Jan 31 [cited 2025 Nov 16];278(5):3257–64. Available from: https://pubmed.ncbi.nlm.nih.gov/12215456/

8. Slebe M, Pouw JEE, Hashemi SMS, Menke-van der Houven van Oordt CW, Yaqub MM, Bahce I. Current state and upcoming opportunities for immunoPET biomarkers in lung cancer. Lung Cancer. 2022 Jul 1;169:84–93.

9. Asao H. Interleukin-2. Reference Module in Biomedical Sciences [Internet]. 2014 Jan 1 [cited 2025 Nov 16]; Available from: https://linkinghub.elsevier.com/retrieve/pii/B9780128012383040587

10. Sabarinathan R. Immunoproteasome as a biomarker for immunotherapy: Immunoproteasome. Nat Rev Cancer [Internet]. 2025 Jan 1 [cited 2025 Nov 16];25(1):6. Available from: https://www.nature.com/articles/s41568-024-00759-7

11. Chen D, Jin C, Dong X, Wen J, Xia E, Wang Q, et al. Pan-cancer analysis of the prognostic and immunological role of PSMB8. Scientific Reports 2021 11:1 [Internet]. 2021 Oct 14 [cited 2025 Nov 16];11(1):20492-. Available from: https://www.nature.com/articles/s41598-021-99724-9

12. Kumar R, Dhaka B, Sahoo S, Jolly MK, Sabarinathan R. Prognostic association of immunoproteasome expression in solid tumours is governed by the immediate immune environment. Mol Oncol [Internet]. 2023 Jun 1 [cited 2025 Nov 16];17(6):1041–59. Available from: /doi/pdf/10.1002/1878-0261.13443

13. Kim Y, Yang H, Lee WS, Cheon J, Sang YB, Kang B, et al. High levels of baseline serum IL-10 are associated with reduced clinical benefit from first-line immune checkpoint inhibitor therapy in advanced renal cell carcinoma. J Cancer [Internet]. 2023 [cited 2025 Nov 16];14(6):935. Available from: https://pmc.ncbi.nlm.nih.gov/articles/PMC10158513/

14. Oft M. IL-10: Master Switch from Tumor-Promoting Inflammation to Antitumor Immunity. Cancer Immunol Res [Internet]. 2014 Mar 1 [cited 2025 Nov 16];2(3):194–9. Available from: /cancerimmunolres/article/2/3/194/467083/IL-10-Master-Switch-from-Tumor-Promoting
